# East meets west: cross-cultural ethnic focussed genetic-counselling

**DOI:** 10.1186/1897-4287-10-S2-A55

**Published:** 2012-04-12

**Authors:** Risha Zia, Lesley Andrews

**Affiliations:** 1 Hereditary Cancer Clinic, Prince of Wales Hospital and Saint George Hospital, NSW, Australia

## 

The South East Sydney Illawarra Area Health Service caters for a large proportion of Sydney’s 50,000 Jewish residents. There is a high level of demand for testing for the BRCA1 and 2 Jewish founder mutations, with 83 tests being done in 2010 through Prince of Wales Hospital.

The team at POW hospital consist of four clinicians and three genetic counsellors. One of the clinicians is of Jewish background and is deeply involved with the Jewish community, seeing most of the patients having JFM testing.

In 2009 the successful applicant for the genetic counsellor position working predominantly with this clinician was of Muslim faith, apparent in her dress. Based on the tensions felt between these two minority groups worldwide, the team were concerned that this may be a barrier to good communication and may result in distress to some Jewish patients. The team consulted Area Employment Services Unit, and it was decided to discuss their concerns with the new counsellor and provide her with an opportunity to resolve any concerns she may have. Together they decided that there would be no changes to standard work practices, but that both the clinician and the genetic counsellor would be alert to any discomfort by the patients and see if there was any negative feedback from them.

54 intakes of Jewish patients by the genetic counsellor via telephone or face to face were done in one year, with patient age ranging from 23-67 years. Excellent rapport was built over the telephone with the patients. Most patients shared personal stories openly (the only issue being time management). With face to face consults, the conversation often started with discussion about the scarf worn by the counsellor, which then moved to the real purpose of the consultation. Most patients were aware that they were to be seen by a Jewish clinician.

It was noted that none of the 54 Jewish patients expressed the desire to be seen solely by the Jewish clinician. No patient expressed concern about being seen by a Muslim counsellor to either the clinician, other team members or hospital services. Several patients required in-depth counselling with the genetic counsellor, leading to five referrals to the oncology psychologist. Their complete disclosure was evidence of the trust they felt in the genetic counsellor. Follow-up phone calls to patients after the clinic appointment were universally pleasant with patients expressing satisfaction with service provided, and often making extremely positive remarks about both the clinician and genetic counsellor. A number openly commented on the excellent service provided by the Muslim counsellor.

## Conclusion

Several factors were key to the success of this cross cultural service provision:

• Discussing the potential for patient distress before employment began, so both staff members felt comfortable to maintain a dialogue on the issue

• Underlying excellent rapport between the clinician and genetic counsellor

• Good communication skills of the genetic counsellor to establish good rapport with the patients.

• Open discussion about the Muslim faith of the counsellor with patients when appropriate

• The needs of patients for genetic services were met by the team.

